# Copy number variations (CNVs) and karyotyping analysis in males with azoospermia and oligospermia

**DOI:** 10.1186/s12920-023-01652-2

**Published:** 2023-09-08

**Authors:** Xing Xin, Peng Xu, Nan Wang, Yi Jiang, Jiaqiao Zhang, Shufang Li, Ying Zhu, Cong Zhang, Long Zhang, Hailong Huang, Ling Feng, Shaoshuai Wang

**Affiliations:** 1 Department of Obstetrics and Gynecology, Tongji Hospital, Tongji Medical College, Huazhong University of Science and Technology, No. 1095 Jiefang Road, Wuhan, 430030 Hubei P.R. China; 2 Department of perinatal laboratory, Tongji Hospital, Tongji Medical College, Huazhong University of Science and Technology, Wuhan, 430030 Hubei P.R. China; 3 Department of Andrology, Tongji Hospital, Tongji Medical College, Huazhong University of Science and Technology, Wuhan, 430030 Hubei P.R. China; 4 Department of Rehabilitation Medicine, Zhongnan Hospital of Wuhan University, Wuhan, 430071 Hubei P.R. China

**Keywords:** Copy number variation (CNV), Karyotyping, Azoospermia, Oligospermia

## Abstract

**Background:**

Considering the essential roles that genetic factors play in azoospermia and oligospermia, this study aims to identify abnormal chromosomes using karyotyping and CNVs and elucidate the associated genes in patients.

**Methods:**

A total of 1157 azoospermia and oligospermia patients were recruited, of whom, 769 and 674 underwent next-generation sequencing (NGS) to identify CNVs and routine G-band karyotyping, respectively.

**Results:**

First, 286 patients were co-analyzed using CNV sequencing (CNV-seq) and karyotyping. Of the 725 and 432 patients with azoospermia and oligospermia, 33.8% and 48.9% had abnormal karyotypes and CNVs, respectively. In particular, 47,XXY accounted for 44.18% and 26.33% of abnormal karyotypes and CNVs, respectively, representing the most frequent genetic aberration in azoospermia and oligospermia patients. Nevertheless, big Y and small Y accounted for 7.46% and 16.67% of abnormal karyotypes, respectively. We also identified high-frequency CNVs-loci, such as Xp22.31 and 2p24.3, in azoospermia and oligospermia patients.

**Conclusion:**

Sex chromosome and autosomal CNV loci, such as Xp22.31 and 2p24.3, as well as the associated genes, such as *VCX* and *NACAP9*, could be candidate spermatogenesis genes. The high-frequency abnormal karyotypes, CNV loci, and hot genes represent new targets for future research.

**Supplementary Information:**

The online version contains supplementary material available at 10.1186/s12920-023-01652-2.

## Background

Infertility is the inability to initiate a pregnancy after more than one year of regular unprotected sexual intercourse [1]. Approximately 15% of couples are infertile, with male factors accounting for more than 50% of cases. The primary cause of male infertility is abnormal spermatogenesis, such as azoospermia, oligospermia, poor sperm motility, and morphological abnormalities [2–4]. Approximately 30% of male infertility cases caused by spermatogenic issues are associated with genetic abnormalities [5]. More specifically, chromosomal abnormalities, chromosomal polymorphisms, sex chromosomal and autosomal microdeletions and microduplications, gene mutations, and mitochondrial genome mutations and deletions that lead to overexpression and deletion negatively impact spermatogenesis, affecting sperm concentration or motility [2, 6–8].

Approximately 10% of male infertility patients are categorized as non-obstructive azoospermia (NOA) due to failure in spermatogenesis. Microdeletions and chromosomal abnormalities of the Y chromosome are the key genetic factors in NOA [9], with incidences of 9.7% and 13% in males with azoospermia, respectively [10]. Chromosomal abnormalities that cause male infertility clinically manifest as azoospermia, oligospermia, teratozoospermia, and globozoospermia. That is, numerical and structural changes in sex chromosomes can cause impaired spermatogenesis, whereas autosomal structural abnormalities, including balanced reciprocal translocations on chromosomes 1, 6, 12, and 22, can also lead to azoospermia or severe oligospermia [11].

Small deletions of the long arm of the Y chromosome (Yq) are not visible through conventional banding karyotype. Nevertheless, deletions in the azoospermia factor (AZF) region of the Yq arm among men manifest as azoospermia and oligospermia. Four spermatogenesis loci have been characterized in the Yq11 head, middle, and terminus, namely, AZFa, AZFb, AZFc, and AZFd [2, 12]. However, more than 4000 genes contribute to human spermatogenesis [13].

The microdeletion and microduplication of chromosomal fragments can be detected by analyzing the CNVs [14]. CNV-seq—based on NGS technology for low-depth whole genome sequencing, which detects chromosomal aneuploidy, chromosomal CNVs over 0.1 Mb, polyploidy, and mosaicism as low as 5% [15, 16]. Moreover, CNV-seq has a low associated cost, short run period, high resolution, high accuracy, and requires a small amount of input DNA. Meanwhile, karyotyping, with a maximum resolution of 3 Mb, is the gold standard for identifying chromosomal abnormalities to detect whole and partial chromosomal aneuploidies, unbalanced translocations, polyploidies, and mosaicism [16, 17].

This study analyzed the NGS-based CNV-seq and karyotyping in males with azoospermia and oligospermia to identify novel microdeletion and microduplication targets, as well as genes and abnormal chromosomal karyotypes associated with azoospermia and oligospermia.

## Materials and methods

### Inclusion criteria

Healthy couples that had not become pregnant after one year of sexual intercourse without contraception, and who showed abnormal semen counts upon semen examination were included in this study.

### Exclusion criteria

Adult males with a history of genital tract obstruction or dysfunction (varicocele and obstructive azoospermia) or congenital defects in the structure of the urogenital system were excluded. Adult male patients who had received radiotherapy for reproductive system carcinoma or chemotherapy for malignancies were also excluded.

### Semen analysis

All sperm samples were tested using standard procedures in the laboratory department of Tongji Hospital in Wuhan, China. Semen samples were collected through masturbation after 3–5 days of abstinence, and semen characteristics were evaluated within 1 h after ejaculation. Semen analysis was performed in accordance with the World Health Organization (WHO) standard protocol for diagnosis of azoospermia (absence of sperm in semen) and oligospermia (sperm counts < 15 × 10^6^ ml^− 1^).

### Study population

We recruited 1157 male infertility patients with azoospermia or oligospermia from the obstetrics and andrology department of Tongji Hospital, Wuhan, China, between February 2017 and July 2019. All CNV-seq and karyotype analyses of infertile male samples were performed at the perinatal laboratory of Tongji Hospital (Wuhan, China).

### Chromosome karyotype analysis of peripheral blood

Approximately 2 ml of peripheral blood was collected in heparin vacutainers for chromosomal karyotype analysis. For each sample, 0.5 ml of peripheral blood was cultured in 5 ml complete medium (RPMI 1640 media + 15% fetal calf serum) for 72 h. The basic cytogenetic method included chromosome harvesting, slide preparation, staining, and banding; subsequently, chromosome number and banding patterns were evaluated. Chromosome preparation involved arresting the cells, harvesting and preparing a single cell suspension, hypotonic treatment, and fixation. The culture process was performed according to standard procedures [18]. G-banded metaphase chromosomes were obtained by treating the cells with trypsin and staining with Giemsa stain. In all cases, at least 30 cells in metaphase were analyzed. In mosaicism-suspected cases, 50 cells were counted. The karyotypes were interpreted according to the International System for Human Cytogenetic Nomenclature (ISHCN) recommendations. An abnormal clone was defined as exhibiting structural aberration, or chromosomal gain in at least two metaphases, and chromosome deletion observed in at least three metaphases.

### Sample preparation and copy number variation sequencing (CNV-seq)

For CNV-seq analysis, approximately 1–2 ml of peripheral blood was collected in EDTA vacutainers and sent to the perinatal laboratory of Tongji Hospital, Wuhan, China. DNA was extracted from the peripheral blood using a DNeasy Blood and Tissue Kit (Qiagen, Germany). The DNA was quantified using a NanoDrop spectrophotometer (Thermo Fisher Scientific, United States) (concentration > 50 ng/µl; OD260/280 > 1.8; OD260/230 > 1.5). DNA samples were sent to Beijing Berry Genomics Co., Ltd., for CNV sequencing using the NextSeq CN500 platform (Illumina Inc.). The annotation and interpretation were carried out based on the guidelines of the American College of Medical Genetics and Genomics. The results were determined by referring to the hg19 version of the human genome and the latest data available from the Database of Genomic Variants (DGV), DECIPHER, Online Mendelian Inheritance in Man (OMIM), University of California Santa Cruz (UCSC), and PubMed.

### Genes involved in CNVs

Microdeletion and microduplication of chromosomal fragments detected through CNV-seq may include changes in genetic function and affect gene expression in testicular or other tissues, leading to spermatogenic failure. Hence, the PubMed public data platform of the National Library of Medicine and National Center for Biotechnology Information (NCBI) through Genome Data Viewer was used to search genes of the CNV loci (https://www.ncbi.nlm.nih.gov/genome/guide/human/).

## Results

### Analysis process

**Fig. 1:**
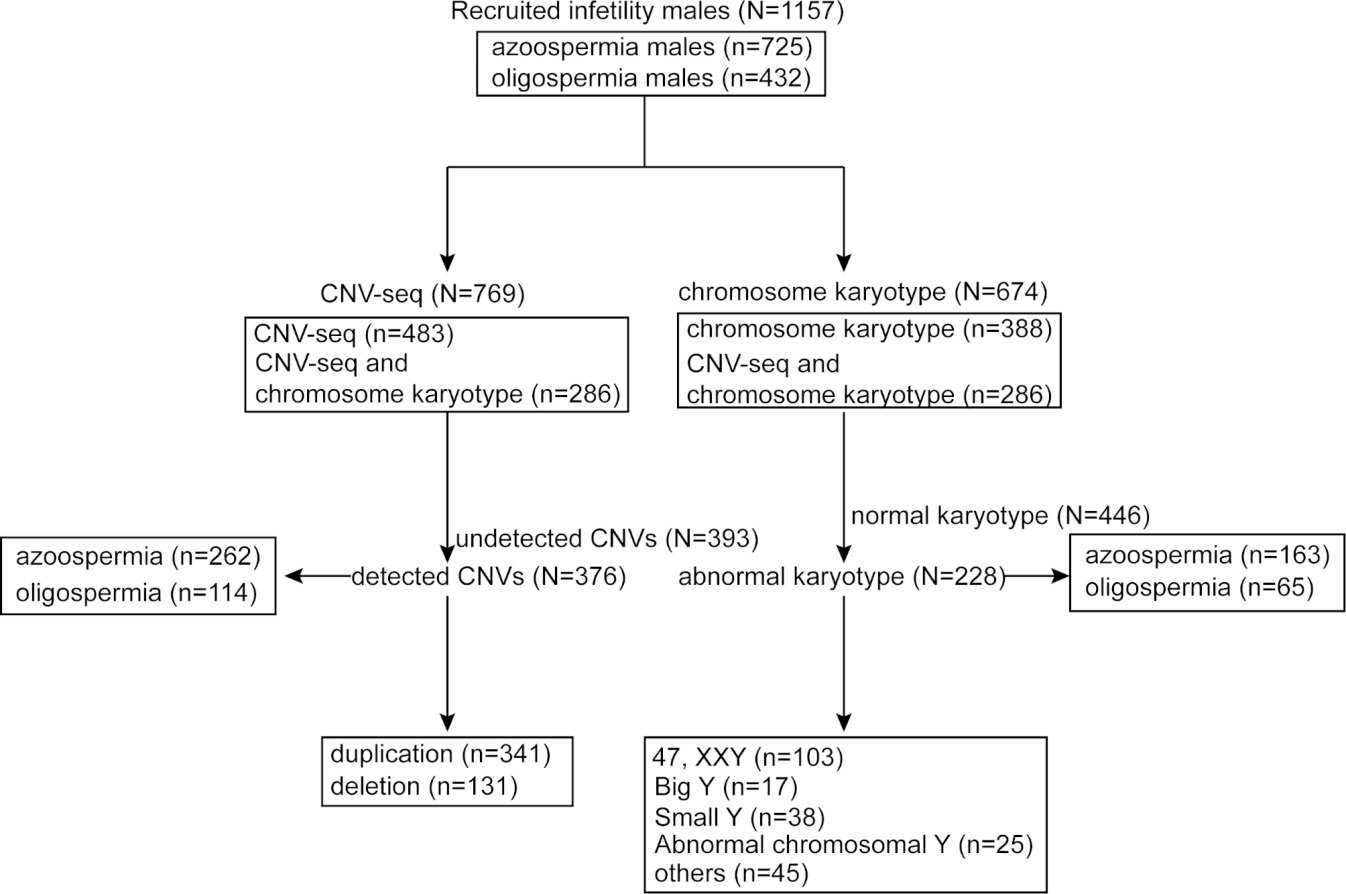
Simplified flow diagram of analysis process in this study

This study recruited 1157 males with infertility, including 725 with azoospermia and 432 with oligospermia. The average ages of patients with azoospermia and oligospermia were 32 ± 0.4 vs. 31 ± 0.6 years (*p* = 0.57), respectively. The simplified analysis process is shown in Fig. 1. Samples for 769 and 674 patients were evaluated via CNV-seq or chromosome karyotyping, respectively, while those for 286 patients were assessed by both methods. Moreover, 26.92% (77/286) of patients had abnormal CNVs and normal chromosome karyotyping, whereas CNVs were not detected in 7.34% (21/286) of patients that had abnormal chromosome karyotyping (Supplemental Table 1).

### Frequency of microduplication and microdeletion CNVs

**Fig. 2:**
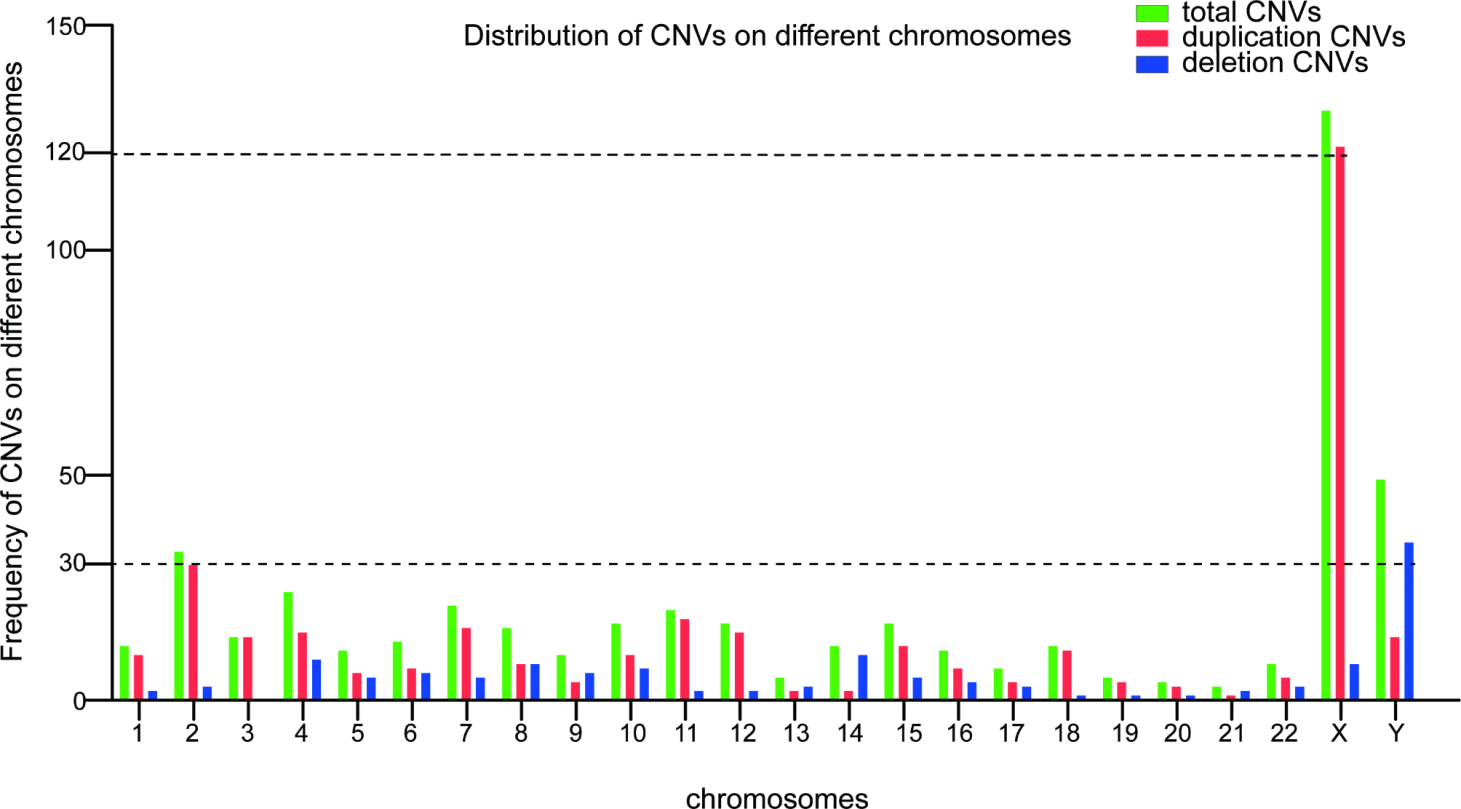
Frequency of CNVs on different chromosomes in males with azoospermia and oligospermia. Green, red, and blue represent total CNVs, microduplication CNVs, and microdeletion CNVs, respective

The frequency of CNVs on different chromosomes is shown in Fig. 2. The highest frequency microduplication CNVs occurred in chromosome X, accounting for 36.07% (123/341) of all CNVs. This was followed by chromosomes 2, 11, 7, 4, 12, and Y, accounting for 8.80% (30/341), 5.28% (18/341), 4.69% (16/341), 4.40% (15/341), 4.40% (15/341), and 4.11% (14/341) of CNVs, respectively.

The highest frequency microdeletion CNVs occurred in chromosome Y, which accounted for 26.72% (35/131) of all CNVs. This was followed by chromosomes 14, 4, X, 8, and 10, accounting for 7.63% (10/131), 6.87% (9/131), 6.11% (8/131), 6.11% (8/131), and 5.34% (7/131) of CNVs, respectively.

Abnormal CNVs were detected in 376 patients, of which 52 carried duplication and deletion CNVs, while 324 patients had either duplication or deletion CNVs. No statistical differences were observed between azoospermia and oligospermia patients in the total deletion and duplication CNVs or in the deletion and duplication CNVs of different chromosomes (*p* > 0.05; Supplementary Table 2).

### Analysis of abnormal chromosomal karyotyping in males with azoospermia and oligospermia

Among the males with infertility, 446/674 cases had normal chromosomal karyotyping, while 228 had abnormal karyotyping. Of these 228 cases, the most frequent abnormal karyotyping was 47,XXY, 103/228 (45.18%) in 100 azoospermia cases and 3 oligospermia cases. Small Y (38/228, 20 azoospermia cases, 18 oligospermia cases), big Y (17/228, 5 azoospermia cases, 12 oligospermia cases), abnormal chromosome Y (25/228), and abnormal chromosome X (2/228) were also of interest. The distribution of abnormal chromosomal karyotyping in azoospermia and oligospermia patients is shown in Supplemental Table 3. The 25 cases with karyotyping related to abnormal chromosome Y are shown in Table 1.

**Table 1:** Distribution of abnormal chromosome Y in males with azoospermia and oligospermia

Chromosomal karyotype	Cases	Azoospermia	Oligospermia
47,XYY	8	3	5
46,X,+mar	2	2	
46, XX	1	1	
mos45,X/46,XY	2	2	
46,XY,inv(Y)(q11, q12)	1	1	
47,XXY,inv(Y)(p11, q11)	1	1	
46,XY,inv(Y)(p11.3, q11.2),add(Y)(p11)	1		1
46,XY,del(Y)(q12)	1	1	
46,XY,del(Y)(q11.21→q21)	1	1	
46,XY,add(Y)(q12)	1		1
46,XY,t(Y,20)(q11, q13.3)	1		1
46,XY,Yqh+	3		3
46,XY,Yqh-	2		2

### High-frequency duplication CNVs

Abnormal CNVs were detected in 376 of the 769 patients. Among these 376, 341 had duplication CNVs, while the others had deletion CNVs (131 patients). Regarding the duplication and deletion CNVs, we focused primarily on chromosomes X, 2, and Y. CNV-seq detected 99 patients with 47,XXY, of which 22 also presented with microduplication and microdeletion of other chromosomes (Supplemental Table 4). Moreover, in the 24 cases of chromosome X microduplication CNVs, 6 cases carried dup(X)(p22.31), 3 dup(X)(p22.33), 2 dup(X)(q21.1), 2 dup(X)(q21.33), 2 dup(X)(q26.3), 2 dup(X)(q27.2), and 2 carried dup(X)(q28). The CNV loci covered genes including *VCX*, *VCX3A*, *PUDP*, *SOX3*, and *HSFX2* (Table 2). The other chromosomal microdeletions and microduplications in chromosome X are presented in Supplemental Table 5. All genes covered by CNV loci are shown in the Supplemental material of Excel 1. Among the 30 cases of chromosome 2 microduplication CNVs, 8 patients carried dup(2)(p24.3), 7 of which had the same NACAP9. Moreover, 3 cases had dup(2)(q32.3) and dup(2)(q37.3), respectively. The CNV loci covered numerous genes, including *ANKRD11P1*, *NRXN1*, *ITSN*, *SLC39A10, HES6, AGXT*, etc. (Table 3). Other chromosomal microdeletion and microduplications accompanied by chromosome 2 aberrations are shown in Supplemental Table 6. All genes covered by CNV loci are presented in the Supplemental material of Excel 2.

**Table 2:** Details regarding 24 males with azoospermia and oligospermia with chromosome X microduplication CNVs and the involved genes

Cases		Chromosomal location	Start-End position	Size (Mb)	Azoospermia	Oligospermia	Number of Genes	CNVs involved genes
1		dup(X)(p11.21)	56,780,001–57,080,000	0.30 Mb		Yes	3	NBDY, et al.
2		dup(X)(p21.1p11.4)	37,440,001–37,980,000	0.54 Mb	Yes		13	PRRG1, et al.
3		dup(X)(p22.31)	7,480,001–7,820,000	0.34 Mb	Yes		0	
4*		dup(X)(p22.31)	6,460,001–7,220,000	0.76 Mb	Yes		6	VCX3A, et al.
5*		dup(X)(p22.31)	7,760,001–8,280,000	0.52 Mb	Yes		6	VCX, et al.
6		dup(X)(p22.31)	6,140,001–6,420,000	0.28 Mb		Yes	3	NLGN4X, et al.
7		dup(X)(p22.31)	6,460,001–7,040,000	0.58 Mb	Yes		4	VCX3A, et al.
8*		dup(X)(p22.31)	6,540,000–7,060,000	0.52 Mb	Yes		3	PUDP, et al.
9*		dup(X)(p22.33)	3,460,001–3,860,000	0.40 Mb	Yes		9	SNORA48B, et al.
		dup(X)(q28)	154,620,000–154,780,000	0.16 Mb			12	CTAG2, et al.
10		dup(X)(p22.33)	3,500,001–3,840,000	0.34 Mb		Yes	8	SNORA48B, et al.
11		dup(X)(p22.33)	3,480,001–3,860,000	0.38 Mb		Yes	9	SNORA48B, et al.
12*		dup(X)(q11.2)	63,220,001–63,420,000	0.20 Mb	Yes		6	SPIN4, et al.
13		dup(X)(q21.1)	77,320,001–78,260,000	0.94 Mb		Yes	15	FGF16, et al.
14		dup(X)(q21.1)	78,860,001–79,160,000	0.30 Mb	Yes		4	P2RY10, et al.
15*		dup(X)(q21.31)	86,280,001–86,380,000	0.10 Mb		Yes	2	DACH2, et al.
16		dup(X)(q21.33)	94,080,001–94,240,000	0.16 Mb	Yes		0	
17		dup(X)(q21.33)	95,120,001–95,520,000	0.40 Mb	Yes		1	CALM1P1.
18*		dup(X)(q25)	122,880,001–123,140,000	0.26 Mb	Yes		1	MRRFP1.
19		dup(X)(q26.3)	133,860,000–134,200,000	0.34 Mb	Yes		9	GPC3, et al.
20*		dup(X)(q26.3)	136,000,001–136,540,000	0.54 Mb	Yes		10	ADGRG4, et al.
21		dup(X)(q27.2)	140,340,001–140,680,000	0.34 Mb		Yes	4	SOX3, et al.
22		dup(X)(q27.2)	140,360,000–140,780,000	0.42 Mb		Yes	6	SOX3, et al.
23		dup(X)(q28)	152,220,000–152,520,000	0.30 Mb	Yes		6	GABRA3, et al.
24		dup(X)(q28)	148,880,000–150,140,000	1.26 Mb		Yes	27	HSFX2, et al.

* represent chromosome X accompanied by others chromosomal microdeletion and microduplication, was showed in supplemental Table 5. All genes of CNVs covered was showed in supplemental material of excel 1 (sheet 1)

**Table 3:** Details regarding 30 males with azoospermia and oligospermia with chromosome 2 microduplication CNVs and the involved genes

Cases	Chromosomal location	Start-End position	Size (Mb)	Azoospermia	Oligospermia	Number of Genes	CNVs involved genes
1	dup(2)(p24.3)	13,540,001–14,340,000	0.80 Mb	Yes		6	NACAP9, et al.
2	dup(2)(p24.3)	13,520,001–14,340,000	0.82 Mb	Yes		7	NACAP9, et al.
3	dup(2)(p24.3)	13,540,001–14,340,000	0.80 Mb		Yes	6	NACAP9, et al.
4	dup(2)(p24.3)	13,540,001–14,340,000	0.80 Mb	Yes		6	NACAP9, et al.
5	dup(2)(p24.3)	13,540,001–14,340,000	0.80 Mb		Yes	6	NACAP9, et al.
6*	dup(2)(p24.3)	13,540,001–14,300,000	0.76 Mb		Yes	6	NACAP9, et al.
7*	dup(2)(p24.3)	13,520,000–14,340,000	0.82 Mb		Yes	7	NACAP9, et al.
8*	dup(2)(p24.3)	13,660,001–14,340,000	0.68 Mb		Yes	4	LINC00276, et al.
9	dup(2)(p12)	81,160,001–81,260,000	0.10 Mb	Yes		1	ANKRD11P1
10	dup(2)(p16.3)	50,980,001–52,200,000	1.22 Mb	Yes		10	NRXN1, et al.
11*	dup(2)(p22.2)	36,820,001–37,280,000	0.46 Mb	Yes		12	STRN, et al.
12	dup(2)(p23.3)	24,340,000–24,560,000	0.22 Mb	Yes		3	ITSN2, et al.
13	dup(2)(p24.1)	23,020,001–23,320,000	0.30 Mb	Yes		3	LOC107985792, et al.
14*	dup(2)(q12.2q12.3)	107,160,001–108,400,000	1.24 Mb	Yes		23	GACAT1, et al.
15*	dup(2)(q12.3)	108,540,001–109,000,000	0.46 Mb		Yes	8	CCDC138, et al.
16*	dup(2)(q13)	110,860,000–110,980,000	0.12 Mb	Yes		2	ACOXL, et al.
17	dup(2)(q14.2)	120,100,001–120,500,000	0.40 Mb	Yes		14	RALB, et al.
18*	dup(2)(q14.3q21.1)	129,280,001–130,060,000	0.78 Mb	Yes		20	RAB6C, et al.
19*	dup(2)(q14.3)	128,880,001–129,240,000	0.36 Mb	Yes		0	-
20*	dup(2)(q21.1q21.2)	132,140,001–132,520,000	0.38 Mb	Yes		17	ZNF285CP, et al.
21*	dup(2)(q32.1)	188,820,001–189,220,000	0.40 Mb	Yes		7	COL3A1, et al.
22	dup(2)(q32.3)	194,200,001–194,860,000	0.66 Mb	Yes		5	LINC01821, et al.
23*	dup(2)(q32.3)	195,640,001–196,160,000	0.52 Mb	Yes		7	SLC39A10, et al.
24	dup(2)(q32.3)	193,620,001–194,080,000	0.46 Mb		Yes	2	SEC61GP1, et al.
25	dup(2)(q34)	210,520,001–211,140,000	0.62 Mb		Yes	4	CPS1, et al.
26*	dup(2)(q35)	216,780,001–217,140,000	0.36 Mb	Yes		12	IGFBP-AS1, et al.
27*	dup(2)(q36.2)	225,880,001–226,000,000	0.12 Mb	Yes		2	LOC105373914, et al.
28	dup(2)(q37.3)	238,120,001–238,620,000	0.50 Mb	Yes		17	HES6, et al.
29*	dup(2)(q37.3)	240,620,001–241,160,000	0.54 Mb		Yes	18	AGXT, et al.
30	dup(2)(q37.3)	238,180,001–238,560,000	0.38 Mb		Yes	14	HES6, et al.

* represent chromosome 2 accompanied by others chromosomal microdeletion and microduplication, was showed in supplemental Table 6. All genes of CNVs covered was showed in supplemental material of excel 2 (sheet 1)

### Deletion and microdeletion CNVs on chromosome Y

A total of 131 deletion and microdeletion CNVs were detected in azoospermia and oligospermia patients. However, herein, we focused on 35 patients with deletions in chromosome Y. Of these, 24 cases had AZF region deletion, including all regions of AZF (5/24), AZFa (5/24), AZFb (6/24), AZFc (2/24), and AZFb + AZFc (6/24). Four cases of chromosome mosaicism of 45, X/46, XY were also detected. The clinical manifestation was azoospermia. Three cases were detected with del(Y)(q11.223), of which two were accompanied by other chromosomal microduplications, namely, dup(20)(p12.1) and dup(7)(q36.2), which manifested as azoospermia or oligospermia, respectively. The CNV loci-covered genes included *NLGN4Y*, *TTTY5*, *DAZ1*, and *USP9Y*. The deletion and microdeletion CNVs and involved genes of chromosome Y are shown in Table 4. All genes covered by CNV loci are shown in the Supplemental material of Excel 3.

**Table 4:** Details of 35 males with azoospermia and oligospermia with chromosome Y microdeletion CNVs and the involved genes

Chromosomal location	Cases	Start-End position	Size (M)	Region of AZF	Azoospermia	Oligospermia	No. of Genes	CNVs involved genes
Y(q11.21)	5			AZFa	Yes			NLGN4Y, et al.
Y(q11.222q11.223)	6			AZFb	Yes			TTTY5, et al.
Y(q11.222q12)	2			AZFc	Yes			DAZ1, et al.
Y(q11.1q12)	2			AZF	Yes			TTTY5, et al.
Y(p11.2q12)	1			AZF	Yes			USP9Y, et al.
Y(q11.21q12)	1			AZF	Yes			DAZ1, et al.
Y(q11.21)	1			AZF	Yes			DAZ1, et al.
Y(q11.222)	5			AZFb + AZFc	Yes			TTTY5, et al.
Y(q11.221)	1			AZFb + AZFc	Yes			TTTY5, et al.
del(Y)(p11.32q12), mos 45, X[60%]/46, XY[40%]	4	1-59,373,566			Yes		589	DAZ1, et al.
Y(p11.2)	1	6,700,001–8,920,000	2.22 M			Yes	52	TTTY12, et al.
Y(q11.21q11.221)	1	14,440,001–15,260,000	0.82 M		Yes		6	NLGN4Y, et al.
Y(q11.223)	1	24,360,001–24,500,000	0.14 M			Yes	5	TTTY17B, et al.
del(Y)(q11.223)*dup(20)(p12.1)	1	24,360,001–24,460,000	0.10 M		Yes		3	RPL41P7, et al.
del(Y)(q11.223)*dup(7)(q36.2)	1	24,360,001–24,520,000	0.16 M		Yes		6	TTTY17B, et al.
Y(p11.2)	1	7,100,001–7,440,000	0.34 M			Yes	6	PRKY, et al.
del(Y)(p11.31p11.2)(mos, 40%)	1	2,640,001–11,200,000	8.56 M			Yes	167	TTTY12, et al.

* represent chromosome Y accompanied by others chromosomal microduplication. All genes of CNVs covered was showed in supplemental material of excel 3 (sheet 1 and sheet 2)

## Discussion

The WHO estimates that male factors have a crucial role in half of all fertility cases [19]. Male infertility is a complex multifactorial pathological condition that presents as azoospermia and oligospermia. The causes of spermatogenic defects are associated with the environment, lifestyle, nutrition, and genetic factors [20]. In fact, in azoospermia, approximately 25% of cases can be associated with genetic factors; however, the genetic factors of male infertility are highly complex [21].

Somatic cells, germ cells, and endocrine signals are essential for the development and maturation of sperm cells. Both sex chromosomal and autosomal genes are involved in the regulation of spermatogenesis. Cytogenetic karyotyping and NGS-based CNV-seq technologies for chromosomal analysis have been widely used in clinical practice. Chromosomal karyotype analysis is the first choice for genetic testing of infertility in males, especially males with azoospermia and oligospermia. The reason is that the karyotype analysis is simple and convenient, with high accuracy and low cost. In this study, Klinefelter’s syndrome (KS, 47,XXY) was identified as a common chromosomal abnormality in males with infertility (103/674, 15.28%). However, a 45,X/46,XY karyotype was also observed in two males with azoospermia. Chromosomal polymorphisms are primarily considered to have no obvious clinical phenotypes, however, other studies have reported that they are related to infertility and recurrent spontaneous abortion (RSA) [6]. Big Y and small Y are considered Y chromosomal polymorphisms associated with RSA. Hence, we speculate that big Y and small Y could be related to azoospermia and oligospermia. Other chromosome Y abnormalities, such as 47,XYY and 46,X,+mar, and structural aberrations of chromosome Y (inversions, translocations, deletions, and additions), as well as Yqh + and Yqh- were also observed in males with azoospermia and oligospermia. We found a rare case of a male with azoospermia who had a 46,XX karyotype. This testicular disorder of sex development is a rare genetic syndrome, causing male infertility due to the absence of the AZF region in chromosome Yq [22].

Sex chromosomes and autosomal genes participate in spermatogenesis regulation [20]. Meanwhile, 47,XXY (KS) is the main genetic factor of male infertility, accounting for 10–15% of oligospermia and NOA cases [23]. In our study, we detected 99 KS patients through CNV-seq, 77 of which were 47,XXY; 72/77 cases showed azoospermia, and 5 exhibited oligospermia. Furthermore, 22 cases (22/99) were 47,XXY accompanied by other chromosomal microdeletions and microduplications, except for one case of 47,XXY with dup(18)(p11.32) which manifested as oligospermia, while the other 21 cases manifested as azoospermia (Supplemental Table 4). Chromosome X is enriched in genes associated with spermatogenesis. Infertile males reportedly have Xp22.31, Xp22.1, Xp11.23, and Xq28 CNV loci [24]. In this study, 24 patients with chromosome X microduplication CNVs were detected, of which 7 were accompanied by other chromosomal microdeletions and microduplications (Table 2). Moreover, 5 of 6 patients with dup(X)(p22.31) exhibited azoospermia. As such, we speculate that the Xp22.31 loci might be associated with male fertility. The regions involved *VCX*, *VCX3A*, *GPC3*, *PUDP*, *SOX3*, and *HSFX2*, among others. X-linked/Y-linked (*VCX/Y*) genes encode a group of proteins expressed exclusively in male germ cells among normal tissue. The family members include *VCX, VCX2, VCX3A, VCX3B, VCY*, and *VCY1B*. All copies of *VCX* and *VCY* are transcribed exclusively in the testis, likely within germ cells [25]. Moreover, the *VCX/Y* protein family might participate in ribosome assembly regulation during spermatogenesis [26]. *VCX* may act by mediating the mitochondria-dependent apoptosis pathway or p53-Bax pathway to prompt cell apoptosis and inhibit cell growth, delaying cell progression in G1 to S transition, and leading to spermatogenesis impairment and failure [27]. Some genes have significance in spermatogenesis, while the functions of others are unknown. For example, *SOX3* is important for regulating embryonic development and shows homology to the *SRY* gene. There is enrichment in male-specific genes on the X chromosome [20]. Therefore, the microdeletion and microduplication of CNVs on the X chromosome are responsible for azoospermia and oligospermia. The severity of the clinical phenotype is related to the length of deletion and duplication bases, the location of the region, and whether other chromosomal microdeletions and microduplications are present. In these patients, we detected that the most frequent autosomal CNVs was microduplication on chromosome 2 (30 cases). Moreover, 7/30 cases had dup(2)(p24.3) (chromosome 2: 13,520,000–14,340,000), involving *NACAP9, LOC105373438*, and *LINC00276*, among others (Table 3). We considered that the CNV loci of 2p24.3 may be associated with spermatogenesis. Additionally, the 2p24.3 location on chromosome 2 (12,000,001–16,500,000) covered many genes, only a portion of which (including *MIR3681HG, MIR3125, LOC100506474, LINC00276, LRATD1, NBAS, DDX1, MYCNUT, MYCNOS, MYCN*, and *GACAT3*) have been previously reported in literature, primarily in association with different cancer types. Meanwhile, only one gene *MYCN* may be associated with male infertility [28]. However, the roles of *NACAP9*, *LINC00276*, and *MYCN* in spermatogenesis require further investigation. Moreover, given that only 2/4 patients with the same dup(2)(p24.3) loci manifested as azoospermia and oligospermia, spermatogenesis might be associated with factors other than genomics.

The microdeletions in the AZF region on chromosome Y are well-characterized causes of male infertility involving azoospermia and oligospermia [9, 13]. The AZF region contains many critical genes associated with spermatogenesis, germ cell development, and spermatocyte maturation, such as *USP9Y*, *KDM5D*, and *DAZ* [29]. Microdeletion loci on chromosome Y, such as Yp11.2, Yq11.21q11.221, and Yq11.223, may be associated with spermatogenesis. Hence, the associated genes, including *NLGN4Y*, *TTTY5*, and *SRY*, require further functional analyses to clarify their relationship with spermatogenesis. With the rapid development of sequencing technology, genomics has provided new tools to better understand the genetics of male infertility. In particular, deletion and microdeletion of the AZF region of chromosome Y have been found to involve genes related to spermatogenesis. Moreover, microdeletion and microduplication of chromosome X, autosomal genes, involved genes, and abnormal chromosomal karyotypes are associated with spermatogenesis and warrant further research.

The limitations of our study include its small sample size and single research center design. Moreover, we did not perform CFTR analysis nor aCGH and Taqman assays to verify detected CNV loci. Hence, since CNV-seq cannot detect single base mutations and certain microdeletions or microduplications, exome sequencing is needed in future studies.

## Conclusion

Various genetic factors contribute to spermatogenic dysfunction. Deletion of the AZF region of the Yq arm primarily manifests as azoospermia and oligospermia. Meanwhile, small Y and big Y may be related to azoospermia and oligospermia. CNV-seq detected that the AZF region of chromosome Y deletion primarily manifested as azoospermia and oligospermia. Various microdeletion and microduplication CNV loci of chromosome Y and the covered genes require further investigation to elucidate their roles in spermatogenesis. Nevertheless, our CNV-seq analysis detected important CNV loci, such as Xp22.31 and 2p24.3, and the associated genes (*VCX3A* and *NACAP9*), which might serve as candidate spermatogenesis CNV loci and hot genes.

### Electronic supplementary material

Below is the link to the electronic supplementary material.

Supplementary Material 1: Excel 1

Supplementary Material 2: Excel 2

Supplementary Material 3: Excel 3

Supplementary Material 4: Table 1

Supplementary Material 5: Table 2

Supplementary Material 6: Table 3

Supplementary Material 7: Table 4

Supplementary Material 8: Table 5

Supplementary Material 9: Table 6

## Data Availability

The data that support the findings of this study are not openly available due to reasons of sensitivity and are available from the corresponding author upon reasonable request. Data are located in controlled access data storage at Prenatal Diagnosis Center of Tongji Hospital.

## List of Abbreviations

CNVs: Copy number variations
CNV-seq: CNV sequencing
NGS: Next-generation sequencing
NOA: Non-obstructive azoospermia
AZF: Azoospermia factor
Yq: Long arm of the Y chromosome
RSA: Recurrent spontaneous abortion
DGV: Database of Genomic Variants
OMIM: Online Mendelian Inheritance in Man
