# Supplementary material for: Copy number variations (CNVs) and karyotyping analysis in males with azoospermia and oligospermia
Source: BMC Med Genomics. 2023 Sep 8;16:213. doi: 10.1186/s12920-023-01652-2 (PMC10485952; doi:10.1186/s12920-023-01652-2)
Supplement: Supplementary file 4 — Supplementary Material 4: Table 1 [file 12920_2023_1652_MOESM4_ESM.docx]

**Supplemental table 1.** The number of people with detected or undetected CNVs and normal or abnormal karyotypes in males with azoospermia and oligospermia.

|  | No CNVs detected | Detected CNVs | Total |
| --- | --- | --- | --- |
| Azoospermia | 234(47.18%) | 262(52.82%) | 496(100%) |
| Oligospermia | 159(58.24%) | 114(41.76%) | 273(100%) |
|  |  |  |  |
|  | Normal karyotype | Abnormal karyotype | Total |
| Azoospermia | 255(61.00%) | 163(39.00%) | 418(100%) |
| Oligospermia | 191(74.61%) | 65(25.39%) | 256(100%) |
|  |  |  |  |
|  | Normal karyotype | Abnormal karyotype | Total |
| No CNVs detected | 127(44.41%) | 21(7.34%) | 148 |
| Detected CNVs | 77(26.92%) | 61(21.33%) | 138 |
| Total | 204 | 82 | 286 |
