# Supplementary material for: Copy number variations (CNVs) and karyotyping analysis in males with azoospermia and oligospermia
Source: BMC Med Genomics. 2023 Sep 8;16:213. doi: 10.1186/s12920-023-01652-2 (PMC10485952; doi:10.1186/s12920-023-01652-2)
Supplement: Supplementary file 5 — Supplementary Material 5: Table 2 [file 12920_2023_1652_MOESM5_ESM.docx]

**Supplemental table 2.** The statistical difference between azoospermia and oligospermia patients in all deletion and duplication CNVs as well as deletion and duplication CNVs of different chromosomes.

|  | Azoospermia | Oligospermia | Total | *p* |
| --- | --- | --- | --- | --- |
| All-del | 43 | 30 | 73 | 0.072 |
| All-dup | 176 | 75 | 251 |  |
| Total | 219 | 105 | 324 |  |
| Chr.X-del | 7 | 2 | 9 | 0.668 |
| Chr.X-dup | 94 | 12 | 106 |  |
| Total | 101 | 14 | 115 |  |
| Chr.Y-del | 7 | 4 | 11 | 0.700 |
| Chr.Y-dup | 9 | 7 | 16 |  |
| Total | 16 | 11 | 27 |  |
| Chr.2-del | 0 | 2 | 2 | 0.128 |
| Chr.2-dup | 17 | 8 | 25 |  |
| Total | 17 | 10 | 27 |  |
| Chr.4-del | 3 | 1 | 4 | 0.946 |
| Chr.4-dup | 11 | 4 | 15 |  |
| Total | 14 | 5 | 19 |  |
| Chr.11-del | 2 | 0 | 2 | 0.486 |
| Chr.11-dup | 7 | 6 | 13 |  |
| Total | 9 | 6 | 15 |  |
| Chr.12-del | 2 | 0 | 2 | 0.524 |
| Chr.12-dup | 8 | 5 | 13 |  |
| Total | 10 | 5 | 15 |  |
| Chr.7-del | 3 | 0 | 3 | 0.505 |
| Chr.7-dup | 7 | 4 | 11 |  |
| Total | 10 | 4 | 14 |  |
| Chr.15-del | 2 | 2 | 4 | 0.853 |
| Chr.15-dup | 5 | 4 | 9 |  |
| Total | 7 | 6 | 13 |  |
| Chr.1-del | 1 | 1 | 2 | 0.887 |
| Chr.1-dup | 5 | 4 | 9 |  |
| Total | 6 | 5 | 11 |  |
| Chr.6-del | 3 | 2 | 5 | 0.819 |
| Chr.6-dup | 4 | 2 | 6 |  |
| Total | 7 | 4 | 11 |  |
| Chr.8-del | 0 | 4 | 4 | 0.491 |
| Chr.8-dup | 2 | 5 | 7 |  |
| Total | 2 | 9 | 11 |  |
| Chr.18-del | 1 | 0 | 1 | 0.327 |
| Chr.18-dup | 6 | 4 | 10 |  |
| Total | 7 | 4 | 11 |  |
| Chr.10-del | 2 | 0 | 2 | 0.315 |
| Chr.10-dup | 6 | 2 | 8 |  |
| Total | 8 | 2 | 10 |  |
| Chr.16-del | 3 | 0 | 3 | 0.167 |
| Chr.16-dup | 2 | 4 | 6 |  |
| Total | 5 | 4 | 9 |  |
| Chr.22-del | 1 | 2 | 3 | 0.676 |
| Chr.22-dup | 1 | 4 | 5 |  |
| Total | 2 | 6 | 8 |  |
| Chr.5-del | 0 | 3 | 3 | 0.100 |
| Chr.5-dup | 3 | 0 | 3 |  |
| Total | 3 | 3 | 6 |  |
| Chr.17-del | 0 | 2 | 2 | 0.400 |
| Chr.17-dup | 2 | 1 | 3 |  |
| Total | 2 | 3 | 5 |  |
| Chr.19-del | 1 | 0 | 1 | 0.400 |
| Chr.19-dup | 1 | 3 | 4 |  |
| Total | 2 | 3 | 5 |  |

Chr: chromosome. del: deletion. dup: duplication.
