# Supplementary material for: Copy number variations (CNVs) and karyotyping analysis in males with azoospermia and oligospermia
Source: BMC Med Genomics. 2023 Sep 8;16:213. doi: 10.1186/s12920-023-01652-2 (PMC10485952; doi:10.1186/s12920-023-01652-2)
Supplement: Supplementary file 6 — Supplementary Material 6: Table 3 [file 12920_2023_1652_MOESM6_ESM.docx]

**Supplemental table 3.** Distribution of abnormal chromosomal karyotype in males with azoospermia and oligospermia.

|  | Cases | Azoospermia | Oligospermia |
| --- | --- | --- | --- |
| 47, XXY | 103 | 100 | 3 |
| Big Y | 17 | 5 | 12 |
| Small Y | 38 | 20 | 18 |
| Abnormal chromosomal Y | 25 | 12 | 13 |
| Abnormal chromosomal X | 2 | 2 |  |
| Others | 43 | 24 | 19 |
