# Supplementary material for: Copy number variations (CNVs) and karyotyping analysis in males with azoospermia and oligospermia
Source: BMC Med Genomics. 2023 Sep 8;16:213. doi: 10.1186/s12920-023-01652-2 (PMC10485952; doi:10.1186/s12920-023-01652-2)
Supplement: Supplementary file 7 — Supplementary Material 7: Table 4 [file 12920_2023_1652_MOESM7_ESM.docx]

**Supplemental table 4.** 47,XXY and of which is accompanied by other chromosomal microduplication and microdeletion.

| Chromosomal location | cases | | azoospermia | oligospermia | |  |
| --- | --- | --- | --- | --- | --- | --- |
| 47, XXY | 77 | | 72 | 5 | |  |
| 47, XXY; dup(1)(q41), 0.36Mb | 1 | | 1 |  | |  |
| 47, XXY; dup(1)(q31.1), 2.58Mb | 1 | | 1 |  | |  |
| 47, XXY; dup(2)(q12.2q12.3), 1.24Mb | 1 | | 1 |  | |  |
| 47, XXY; dup(2)(q14.3q21.1), 0.78Mb | 1 | | 1 |  | |  |
| 47, XXY; dup(3)(p26.1), 0.46Mb | 1 | | 1 |  | |  |
| 47, XXY; del(4)(q28.3), 0.46Mb | 1 | | 1 |  | |  |
| 47, XXY; dup(4)(q28.1), 0.46Mb; dup(5)(q21.2q21.3), 0.98Mb | 1 | | 1 |  | |  |
| 47, XXY; dup(5)(p12-p11), 0.36Mb | 1 | | 1 |  | |  |
| 47, XXY; del(5)(p13.3), 0.36Mb | 1 | | 1 |  | |  |
| 47, XXY; dup(6)(q14.1),0.26Mb | 1 | | 1 |  | |  |
| 47, XXY; del(9)(p23), 0.34Mb | 1 | | 1 |  | |  |
| 47, XXY; del(10)(q23.1), 0.36Mb | 1 | | 1 |  | |  |
| 47, XXY; dup(11)(q11), 0.70Mb; del(4)(q35.2), 2.22Mb | 1 | | 1 |  | |  |
| 47, XXY; dup(12) (p13.32), 0.58Mb; dup(12)(p13.31), 0.52Mb | 1 | | 1 |  | |  |
| 47, XXY; del(14)(q11.2), 0.40Mb | 1 | | 1 |  | |  |
| 47, XXY; dup(15)(q26.3),0.38Mb | 1 | | 1 |  | |  |
| 47, XXY; dup(17)(p11.2-p11.1), 0.58Mb | 1 | | 1 |  | |  |
| 47, XXY; dup(18)(p11.31-p11.23), 0.50Mb; dup(20)(q13.12), 0.42Mb | 1 | | 1 |  | |  |
| 47, XXY; dup(18)(p11.32), 0.32Mb | 1 | |  | 1 | |  |
| 47, XXY; del(Y)(q11.21),15.58Mb | 1 | | 1 |  | |  |
| 47, XXY; del(Y)(q11.1q12), 15.70Mb | 1 | 1 | | |  | |
| 47, XXY; del(Y)(q11.1q12), 15.70Mb | 1 | 1 | | |  | |
