# Supplementary material for: Copy number variations (CNVs) and karyotyping analysis in males with azoospermia and oligospermia
Source: BMC Med Genomics. 2023 Sep 8;16:213. doi: 10.1186/s12920-023-01652-2 (PMC10485952; doi:10.1186/s12920-023-01652-2)
Supplement: Supplementary file 8 — Supplementary Material 8: Table 5 [file 12920_2023_1652_MOESM8_ESM.docx]

**Supplemental table 5.** Others chromosomal microdeletion and microduplication accompanied with chromosome X.

|  | Chromosomal location | Start-End position | Size (Mb) | Number of Genes | CNVs involved genes |
| --- | --- | --- | --- | --- | --- |
| 4* | dup2q32.1 | 188820001-189220000 | 0.40Mb | 7 | COL3A1, et al |
|  | dup7q34 | 141720001-141980000 | 0.26Mb | 20 | WEE2, et al |
| 5* | dup(16)(p13.11) | 15040001-16260000 | 1.22 Mb | 31 | PDXDC1, et.al |
| 8* | dup(2)(q32.3) | 195640001-196160000 | 0.52Mb | 8 | SLC39A10, et al |
| 12* | dup(Y)(q11.221) | 16160000-16500000 | 0.34Mb | 2 | RNU6-109P, et al |
| 15* | del(X)(p21.1) | 35620001-36260000 | 0.64Mb | 8 | SIAH1P1, et al |
| 18* | del(4)(p16.3) | 40001-260000 | 0.22Mb | 6 | ZNF595, et al |
| 20* | del14q21.2 | 45700001-45960000 | 0.26Mb | 3 | LINC02303, et al |

* represent chromosome X accompanied by others chromosomal microdeletion and microduplication. All genes of CNVs covered was showed in supplemental material of excel 1 (sheet 2).
