# Supplementary material for: Copy number variations (CNVs) and karyotyping analysis in males with azoospermia and oligospermia
Source: BMC Med Genomics. 2023 Sep 8;16:213. doi: 10.1186/s12920-023-01652-2 (PMC10485952; doi:10.1186/s12920-023-01652-2)
Supplement: Supplementary file 9 — Supplementary Material 9: Table 6 [file 12920_2023_1652_MOESM9_ESM.docx]

**Supplemental table 6.** Others chromosomal microdeletion and microduplication accompanied with chromosome 2.

| Cases | Chromosomal location | Start-End position | Size (Mb) | Number of Genes | CNVs involved genes |
| --- | --- | --- | --- | --- | --- |
| 6* | dup(6)(q22.31) | 123120000-123300000 | 0.18Mb | 1 | TRDN |
| 7* | del(10)(q21.3) | 68260000-68560000 | 0.3Mb | 10 | SLC25A16, et al. |
| 8* | dup(11)(p11.12) | 49760001-50440000 | 0.68Mb | 27 | OR4A1P, et al. |
| 11* | dup(11)(p11.12) | 50320001-50720000 | 0.40Mb | 2 | LINC02750, et al. |
| 14* | 47, XXY |  |  |  |  |
| 15* | dup(7)(q36.1q36.2) | 152440001-153360000 | 0.92Mb | 14 | ATP5PBP3, et al. |
| 16* | dup(12)(q11q12) | 37980001-38440000 | 0.46Mb | 10 | AK6P2, et al. |
| 18* | 47, XXY |  |  |  |  |
| 19* | dup(5)(q21.1) | 99440001-99720000 | 0.28Mb | 3 | GUSBP8, et al. |
| 20* | del(7)(q11.21) | 64680001-65180000 | 0.50Mb | 25 | ZNF273, et al. |
| 21* | dup(X)(p22.31) | 6460001-7220000 | 0.76Mb | 7 | VCX3A, et al |
|  | dup(7)(q34) | 141720001-141980000 | 0.26Mb | 21 | WEE2, et al. |
| 23* | dup(X)(p22.31) | 6540000-7060000 | 0.52Mb | 4 | PUDP, et al. |
| 26* | dup(5)(q21.1) | 99440000-99740000 | 0.30Mb | 3 | GUSBP8, et al. |
|  | del(21)(q22.11) | 32080001-32380000 | 0.30Mb | 8 | MRAP, et al. |
| 27* | dup(10)(p12.1) | 26240001-26380000 | 0.14Mb | 1 | GAD2 |
|  | dup(15)(q12) | 25840001-26420000 | 0.58Mb | 12 | ATP10A, et al. |
| 29* | del(16)(p12.2) | 21940001-22380000 | 0.44Mb | 17 | PDZD9, et al. |

* represent chromosome 2 accompanied by others chromosomal microdeletion and microduplication. All genes of CNVs covered was showed in supplemental material of excel 2 (sheet 2).
